# Exploring Alzheimer’s Awareness: A Content Quality and Reliability Assessment

**DOI:** 10.7759/cureus.93705

**Published:** 2025-10-02

**Authors:** Pracruti J Iyer, Akanksha Soni, Naima Waheed, Elias Abboud, Arva Deesawala, Ruchira Clementina, Mohammed Hazir, Prachi Chunawala, Chunawala Purvi Jatin, Sarayu Vejju, Vaishnavi K, Harshitha Gedda, Bhavya Vats, Swati G Devella, Yagnik Reddy, Usman Khan

**Affiliations:** 1 Medicine, BKL Walawalkar Rural Medical College, Chiplun, Maharashtra, Mumbai, IND; 2 Acute Medical Unit, Stockport NHS Foundation Trust, Stockport, GBR; 3 Medicine, Alfaisal University, Riyadh, SAU; 4 Internal Medicine, Saint Joseph University of Beyrouth, Beyrouth, LBN; 5 Medicine, Research MD, Vadodara, IND; 6 Medicine, Government Medical College, Nizamabad, IND; 7 Medicine, Jawaharlal Institute of Postgraduate Medical Education and Research, Puducherry, IND; 8 General Medicine, All India Institute of Medical Sciences (AIIMS), Rajkot, IND; 9 Medicine, Monroe Township High School, Monroe Township, USA; 10 Medicine, Sapthagiri Institute of Medical Science and Research Centre, Bengaluru, IND; 11 Medicine and Surgery, Sri Padmavathi Medical College for Women, Tirupati, IND; 12 Medicine, DY Patil University, Mumbai, IND; 13 Medicine, Kempegowda Institute of Medical Sciences, Bangalore, IND; 14 Medicine, Prathima Institute of Medical Sciences, Karimnagar, IND; 15 Medicine, Akhtar Saeed Medical and Dental College, Lahore, PAK

**Keywords:** alzheimer's dementia, alzheimer’s dementia, alzheimer’s diseases, neurology, reliability score

## Abstract

Alzheimer’s disease (AD) is a progressive neurodegenerative disorder with significant public health implications. Social media platforms like Instagram have emerged as influential tools for health communication, yet the quality and reliability of content remain variable. This study aimed to systematically evaluate the nature, accuracy, reliability, and overall educational quality of AD-related content disseminated on Instagram, to determine whether the information provided is evidence-based, appropriately sourced, and suitable for public health education. A cross-sectional observational study was conducted over 20 days in January 2025, analyzing posts from six popular Alzheimer’s-related hashtags. A total of 600 posts were screened, of which 288 met the inclusion criteria. Posts were assessed for type, uploader category, content category, factual accuracy, Global Quality Score (GQS), and Reliability Score. Statistical comparisons were made between two groups of posts based on content accuracy and quality. Among the 288 posts analyzed, 162 (56.25%) were images and 126 (43.75%) were videos. Doctors (90, 30.94%) and researchers (82, 28.47%) were the most common uploaders. The majority of posts focused on etiology (66, 22.19%), prevention (60, 20.88%), and symptoms (60, 20.88%). Only 120 (51.9%) posts in Group A were factually accurate, compared to 22 (38.59%) in Group B (*P* < 0.000001). The mean GQS and Reliability Score were significantly higher in Group A (2.28 ± 1.04 and 2.29 ± 1.21) than in Group B (1.93 ± 1.10 and 1.72 ± 1.09), indicating better quality and trustworthiness of content.

Instagram serves as a widely used platform for disseminating Alzheimer’s-related information. However, the overall quality and reliability of content are suboptimal, with less than half of the posts being factually accurate. Healthcare professionals and researchers tend to produce more credible content, while posts from patients and non-medical users are less reliable. There is a pressing need to promote the creation and amplification of evidence-based, high-quality content to enhance public understanding and support for AD.

## Introduction

Alzheimer's disease (AD), an irreversible neurodegenerative disease, is the most common form of dementia in the world, affecting approximately 50% to 70% of all dementia cases [1]. The continuous increase in prevalence, mainly due to an aging population, has made the disease a global health concern recognized by the World Health Organization (WHO). 

According to studies, the number of AD patients is expected to reach 152 million by 2050 [2]. This burdensome disease manifests via progressive cognitive impairment, causing gradual deterioration of episodic memory, linguistic and visual deficiencies, as well as behavioral changes [3]. Hence, the impact on daily life activities is remarkable, inciting prevention measures and awareness actions that can be displayed on social media platforms such as Instagram, YouTube, Facebook, etc. In fact, in this era of technological advancements, social media platforms have become an everyday tool used for different purposes. As these platforms evolve and expand across the world, reaching all communities, notably the healthcare sector, a massive number of people are referring to these media networks to seek health-related information [4]. 

Instagram, a social media platform known for its efficiency, cost-effectiveness, accessibility, visuals, and dynamic interactions, is widely utilised for spreading awareness on several pathologies and health conditions among the population, in particular, AD. However, the unsupervised online content of this application raises questions about the quality, dependability, and reliability of the information flowing through this image-based platform. Also, there is a limited understanding of Instagram as a source of information on AD since no research was conducted discussing the benefits as well as the drawbacks of social media for raising awareness regarding Alzheimer's. Therefore, the purpose of this study is to conduct a content analysis of Alzheimer’s-related posts on Instagram by describing their content characteristics, evaluating the information shared, and using the Global Quality Score (GQS) and Reliability Score to assess their quality and reliability.

## Materials and methods

A cross-sectional observational study was conducted virtually over a span of 10 days in January 2025 to evaluate the nature and quality of content related to AD on Instagram. Given the platform’s widespread use among young and middle-aged adults, Instagram was selected as the medium for data collection to assess the accuracy, credibility, and engagement of posts related to Alzheimer’s awareness and education.

Six commonly used hashtags were identified and included in the study: #Alzheimerawareness, #Alzheimer, #Alzheimerdisease, #Alzheimerprevention, #memoryloss, and #dementia. Only posts in the English language that contained relevant educational, clinical, or experiential information about AD were considered. Posts in other languages or those lacking relevance to the disease were excluded. Each investigator was assigned one hashtag and systematically analyzed 10 *recent* posts per day over the 20-day study period, yielding an initial pool of 600 posts. After applying inclusion and exclusion criteria, 288 posts were finalized for detailed evaluation.

A pre-designed assessment form was used to evaluate each post based on content type, uploader category, nature of information, and factual accuracy. In addition, the educational quality and reliability of each post were evaluated using the GQS and a standardized Reliability Score, respectively (Tables 1-2).

**Table 1:** Global Quality Score (GQS). Source: [5].

GQS	Score
Poor quality, poor flow of the site, most information missing, not at all useful for patients	1
Generally poor quality and poor flow, some information listed, but many important topics are missing, of very limited use to patients	2
Moderate quality, suboptimal flow, some important information is adequately discussed, but others are poorly discussed, somewhat useful for patients	3
Good quality and generally good flow, most of the relevant information is listed, but some topics are not covered, useful for patients	4
Excellent quality and excellent flow, very useful for patients	5

**Table 2:** Reliability Score. Each criterion was scored as 1 (Yes) or 0 (No), with total scores representing the overall reliability of the content. Source: [6].

Evaluation criteria
Are the aims clearly stated and achieved?
Are credible sources of information cited?
Is the content balanced and unbiased?
Are references or additional sources provided for further reading?
Does the post acknowledge areas of uncertainty?

All data were systematically recorded using Microsoft Excel (Microsoft® Corp., Redmond, WA) and analyzed using IBM SPSS Statistics (IBM Corp., Armonk, NY). Descriptive statistics were used to summarize the findings, and inferential analyses (including *Z*-tests and *P*-values) were conducted to determine the statistical significance of differences in content quality and reliability among uploader categories.

## Results

A total of 600 Instagram posts were initially screened across six Alzheimer’s-related hashtags. After applying inclusion and exclusion criteria, 288 posts were included in the final analysis. The distribution of posts per hashtag is presented in Table 3.

**Table 3:** Hashtags and number of posts included.

Hashtag	Initial posts	Final posts
#Alzheimerawareness	100	41
#Alzheimer	100	79
#Alzheimerdisease	100	57
#Alzheimerprevention	100	55
#memoryloss	100	18
#dementia	100	38
Total	600	288

A total of 288 health-related posts were analyzed, comprising 162 images (56.25%) and 126 videos (43.75%).

In terms of uploader type, the majority of content was shared by doctors (90, 30.94%), followed by researchers (82, 28.47%), and healthcare workers (59, 20.46%). Contributions from NGOs (16, 5.5%), relatives of patients (21, 7.2%), patients (9, 3.1%), and pharmaceutical agencies (9, 3.1%) were relatively limited. Only 2 posts (1.23%) came from other sources (Table 4).

**Table 4:** Distribution of content type, uploader type, information category, GQS, and Reliability Score among included Instagram posts (N = 288). Descriptive statistics only; values shown as frequency (*n*) and percentage (%).

Category	Subcategory	*n* (%)
Content type	Images	162 (56.25%)
	Videos	126 (43.75%)
Uploader type	Doctors	90 (30.94%)
	Healthcare workers	59 (20.46%)
	Researchers	82 (28.47%)
	NGOs	16 (5.5%)
	Relatives of patients	21 (7.2%)
	Patients	9 (3.1%)
	Pharmaceutical agencies	9 (3.1%)
	Others	2 (1.23%)
Nature of information shared	Etiology/Causes	66 (22.19%)
	Screening/Symptoms	60 (20.88%)
	Prevention	60 (20.88%)
	Rehabilitation	52 (18.05%)
	Treatment	28 (9.7%)
	Patient experience	9 (3.1%)
	Mortality	3 (1.04%)
	Others	12 (4.16%)
Global Quality Score (GQS)	Very low (1)	105 (36.4%)
	Low (2)	77 (26.7%)
	Medium (3)	56 (19.4%)
	High (4)	40 (13.8%)
	Very high (5)	10 (3.4%)
Reliability Score	Very low (1)	117 (40.6%)
	Low (2)	63 (21.8%)
	Medium (3)	58 (20.1%)
	High (4)	39 (13.5%)
	Very high (5)	11 (3.8%)

Regarding the nature of information shared, the most frequent themes were etiology/causes (66, 22.19%), screening and symptoms (60, 20.88%), and prevention (60, 20.88%), followed by rehabilitation (52, 18.05%). Posts related to treatment (28, 9.7%), patient experiences (9, 3.1%), and mortality (3, 1.04%) were relatively less represented. A small portion (12, 4.16%) addressed other topics.

The quality of content, assessed using the GQS, was found to be predominantly low. About 105 (36.4%) posts scored very low (GQS 1) and 77 (26.7%) scored low (GQS 2). Only 56 (19.4%) achieved a medium score (GQS 3), while 40 (13.8%) and 10 (3.4%) were classified as high and very high, respectively.

Similarly, the reliability of information was limited. A substantial proportion (117, 40.6%) fell into the very low reliability category, followed by 63 (21.8%) low, 58 (20.1%) medium, 39 (13.5%) high, and only 11 (3.8%) very high reliability.

Overall, while doctors and researchers were the dominant contributors, the majority of the shared content demonstrated low quality and low reliability, with preventive and etiological information being most common, whereas patient-centered and treatment-related content remained scarce (Table 5).

**Table 5:** Engagement and reach.

Metric	Value
Total likes	301,498
Total comments	9,478
Total followers of uploaders	1,726,422
Digitally created videos/images	158
Total reach/views	1,126,208

The posts demonstrated significant audience engagement. Collectively, they accumulated 301,498 likes and 9,478 comments, with uploaders collectively having 1.7 million followers. The content reached an estimated 1,126,208 viewers, of which 158 were digitally created videos or images, highlighting the growing role of creative media in health communication. 

The analysis of uploader type with respect to quality and reliability of shared content revealed notable variations (Table 6).

**Table 6:** Uploader-wise GQS and Reliability Scores Scores expressed as median (range). Non-parametric comparisons were descriptive only; no inferential testing was applied at the subgroup level.

Uploader type	GQS score (Median (Range))	Reliability Score (Median (Range))
Doctors	4 (3-4)	3 (3-4)
Healthcare workers	3 (2-4)	3 (1-4)
Researchers	3 (2-4)	3 (2-4)
NGOs	1 (1-2)	2 (1-3)
Relatives of patients	1 (1-2)	1 (1-1)
Patients	1 (1-2)	1 (1-1)
Pharmaceutical agencies	1 (1-2)	2 (1-3)
Others	3 (2-3)	1 (1-1)

Doctors achieved the highest scores, with a median GQS of 4 (range: 3-4) and a Reliability Score of 3 (range: 3-4), indicating generally high-quality and reliable information. Healthcare workers and researchers followed, both with a median GQS of 3 (range: 2-4) and a Reliability Score of 3, suggesting moderate quality and consistency.

In contrast, content uploaded by NGOs showed a median GQS of 1 (range: 1-2) and a reliability score of 2 (range: 1-3), reflecting limited quality but slightly better reliability compared to patient-generated or relative-generated posts. Posts shared by relatives of patients and patients themselves had the lowest values, with both reporting a median GQS of 1 (range: 1-2) and a reliability score fixed at 1, highlighting minimal informational credibility.

Similarly, pharmaceutical agencies demonstrated low quality (median GQS 1; range: 1-2) with slightly higher reliability (median 2; range: 1-3). The “Others” category showed a median GQS of 3 (range: 2-3) but poor reliability (median 1; range: 1-1).

Overall, the findings indicate that while doctors provided the most reliable and higher-quality content, posts generated by patients, relatives, and pharmaceutical agencies were characterized by poor quality and low reliability, underlining substantial variability depending on the uploader type.

A total of 288 posts were classified and assessed for accuracy. In Group A, out of 231 posts, 120 (51.9%) were factually correct. In comparison, Group B had 57 posts, of which 22 (38.59%) were correct (Table 7).

**Table 7:** Classification of posts - accuracy. Values compared using the *Z*-test for proportions. *P < 0.05 is considered statistically significant.

Group	Total posts (*n*)	Correct posts (*n*)	Percentage	*Z*-value	*P*-value
Group A	231	120	51.9%	1.90	<0.001*
Group B	57	22	38.59%		

A *Z*-test for proportions demonstrated a statistically significant difference (*Z* = 1.90, *P* < 0.001), indicating that Group A posts were overall more factually accurate than Group B

The mean GQS was significantly higher in Group A (2.28 ± 1.04) compared to Group B (1.93 ± 1.10), with t(286) = 2.17, *P* = 0.031.

Similarly, the mean Reliability Score of Group A (2.29 ± 1.21) was greater than that of Group B (1.72 ± 1.09), showing a highly significant difference (t(286) = 3.46, *P* < 0.001).

These findings indicate that Group A posts were superior in both quality and reliability (Table 8).

**Table 8:** Comparison of GQS and Reliability Scores between groups. An independent samples t-test is used for mean comparisons. *P < 0.05 is considered statistically significant. SD, standard deviation

Parameter	Group A (Mean ± SD)	Group B (Mean ± SD)	t-value	*P*-value
Global Quality Score (GQS)	2.28 ± 1.04	1.93 ± 1.10	t(286) = 2.17	0.031*
Reliability Score	2.29 ± 1.21	1.72 ± 1.09	t(286) = 3.46	<0.001*

## Discussion

This study analyzed Instagram posts containing the hashtags #Alzheimerawareness, #Alzheimer, #Alzheimerdisease, #Alzheimerprevention, #memoryloss, and #dementia. The 288 posts included in this analysis collectively reached a large audience, as evidenced by the 1,726,422 followers associated with the uploading accounts. Public engagement was notable, with 301,498 likes and 9,478 comments, reflecting strong interest and interaction, as shown in the analysis. Over half of these posts were image-based, while the remaining were video content. Given that approximately 45% of the global population uses social media [7], platforms like Instagram have become prominent sources of health-related information, especially through visually engaging content [4].

In this study, 90 (30.94%) posts were uploaded by doctors, followed by researchers (82, 28.47%) and healthcare workers (59, 20.46%), as summarized in the analysis. Evidence suggests that healthcare professionals are increasingly using platforms like Instagram to connect with patients and share their insights [8-10], contributing to the digital dissemination of reliable health information - a phenomenon known as *e-professionalism* [11].

Researchers also play a vital role in spreading evidence-based knowledge, especially in complex and chronic conditions such as AD, which accounts for around 60% of all dementia cases and is currently irreversible [12]. Their presence on social media contributes significantly to public education and disease awareness [13].

Conversely, 9 (3.1%) posts were shared by patients and 21 (7.2%) by relatives of individuals with AD. Thus, over 10% of the analyzed posts represent personal narratives and lived experiences. These accounts often highlight the daily burden of the disease and coping strategies. According to a 2021 systematic review on health-related social media use, many patients turn to online platforms to seek advice, share stories, and learn from others with similar experiences to guide lifestyle changes and explore treatment options [14].

Among organizations, NGOs accounted for 5.5% (*n* = 16) of the content, while pharmaceutical companies contributed 3.1% (*n* = 9). These findings suggest the need for broader involvement of non-profits and advocacy groups in social media health education. At the same time, caution is advised regarding the presence of pharmaceutical companies, as their content may blur the line between education and promotion [15].

Content analysis revealed that 66 (22.19%) posts focused on etiology, while 60 (20.88%) discussed screening/symptoms, and another 60 (20.88%) promoted prevention. These findings align with those of Gomaa et al. in their study *Skin Cancer Narratives on Instagram*, where over 30% of posts addressed prevention and tumor characteristics [16]. Thus, Instagram appears to be a valuable platform for raising disease awareness and emphasizing early detection and prevention.

As shown in the analysis, the highest GQS and Reliability Scores were consistently observed in posts by doctors, healthcare professionals, and researchers. In fact, 36.6% of posts scored ≥3 on the GQS, and 37.4% received reliability scores of ≥3. Comparing uploader groups, those in the medical field (Group A) shared 120 posts (51.9%) that were factually correct, compared with 22 posts (38.59%) from non-medical sources (Group B), indicating a significant disparity in content credibility.

With the growing reliance on social media for health information, the concern over misinformation has become increasingly relevant. This trend explains why more healthcare professionals are actively participating in social media discourse - to correct false narratives and deliver verified guidance [9]. Future research should address several limitations of the present study. Since the analysis was restricted to posts with selected hashtags on Instagram at a single point in time, future studies could adopt a broader, longitudinal approach across multiple social media platforms to capture evolving trends and ensure greater generalizability. Moreover, reliance on likes and comments as indicators of engagement may not fully reflect actual knowledge gain or behavioral change; therefore, future work should incorporate audience surveys or outcome-based measures to assess impact more comprehensively.

## Conclusions

This study highlights the growing use of Instagram as a medium for Alzheimer’s awareness and education. While the platform provides significant opportunities for outreach and public engagement, the accuracy and reliability of the information shared remain inconsistent. Content posted by healthcare professionals and researchers generally demonstrated higher educational quality and factual correctness compared to posts from patients, relatives, and non-medical users.

These findings emphasize the urgent need for active involvement of health professionals and credible institutions in disseminating content on social media platforms. Ensuring that information is both accessible and evidence-based requires improved content regulation, enhanced digital health literacy, and the promotion of verified medical communication. Collectively, these measures are essential to foster responsible health information sharing in the digital age.
